# ECG Changes in a Patient Presenting With Chest Pain Secondary to Left-Sided Primary Spontaneous Pneumothorax: A Case Report-Based Literature Review

**DOI:** 10.7759/cureus.33904

**Published:** 2023-01-18

**Authors:** Zahid Khan

**Affiliations:** 1 Acute Medicine, Mid and South Essex NHS (National Health Services) Foundation Trust, Southend on Sea, GBR; 2 Cardiology, Bart’s Heart Centre, London, GBR; 3 Cardiology and General Medicine, Barking, Havering and Redbridge University Hospitals NHS (National Health Services) Trust, London, GBR; 4 Cardiology, Royal Free Hospital, London, GBR

**Keywords:** emergency chest drain, chest xray cx-ray, chest tube, electrocardiogram (ecg/ekg), primary pneumothorax, shortness of breath (sob), chest pain in the young, right axis deviation

## Abstract

Pneumothorax is the accumulation of air in the extrapulmonary space between the pleura and the chest wall. Spontaneous pneumothorax can present with various electrocardiographic (ECG) findings including axis deviation, bundle branch block and T waves inversion. A 23-year-old young male patient of slim build presented to the accident and emergency department with sudden-onset chest pain and shortness of breath. He had pleuritic chest pain, worse on breathing. Electrocardiogram showed right axis deviation, diminished or low-amplitude R waves and small-amplitude QRS complexes in the precordial leads. Vital signs were stable and physical examination showed reduced air entry on the left side. Chest radiography showed significant left-sided pneumothorax and the patient had an emergency chest drain inserted. ECG changes resolved with the resolution of pneumothorax. He was discharged home after four days of hospital admission and complete resolution of pneumothorax.

## Introduction

Pneumothorax is the extra-pulmonary collection of air within the pleural space between the lungs and the chest wall [1]. It can be either primary or secondary pneumothorax. Primary spontaneous pneumothorax (PSP) can present in young patients and may mimic cardiac events [1]. Primary pneumothorax that acquires tension physiology usually requires surgical intervention as it becomes a life-threatening emergency [2]. It is a frequently encountered problem in the acute medical practice and can happen in young patients with no medical problems as well as in patients with respiratory problems such as chronic obstructive pulmonary disease (COPD) and asthma. Patients may present with chest pain, dyspnoea, tachycardia and tachypnoea. Electrocardiogram (ECG) may show certain features that are associated with spontaneous primary and tension pneumothorax. These ECG changes are not widely appreciated although they can be of significant diagnostic aid as chest examination may not always be reliable in particular in patients with COPD and other respiratory conditions. Several electrocardiographic findings have been documented previously for both right- and left-sided pneumothorax. ECG features in right-sided pneumothorax include T-wave inversion and right bundle branch block [3]. Left-sided pneumothorax has also been reported to cause a decrease in QRS amplitude in all the chest leads except V1, 3, and 4 [3]. Right axis deviation (RAD) can be a normal variation in children and young adults. It can also be associated with limb-lead reversal, right ventricular hypertrophy, conduction defects such as left posterior fascicular block and right bundle branch block, myocardial infarction, Wolff-Parkinson-White syndrome, dextrocardia, left pneumothorax, and conditions causing right ventricular strain (e.g., pulmonary embolism, pulmonary hypertension, chronic lung disease and resultant cor-pulmonale) [4]. We present a case of a young male patient presenting with sudden-onset chest pain and the ECG showed RAD. Chest radiography confirmed left-sided pneumothorax. The prominent ECG changes associated with left-sided pneumothorax include RAD, clockwise rotation of the transition zones, inverted T-waves and diminution of QRS-wave amplitude in the precordial leads [1]. These changes can be explained primarily by the changes in the anatomical position of the heart within the thoracic cavity and the possible mechanisms include factors affecting electrical impulses; the clockwise rotation of the heart, the rightward shift of the mediastinum, and the accumulation of extra-pulmonary air between the heart and the electrodes [1]. ECG changes associated with right-sided pneumothorax on the other hand are poorly understood. Previous case reports have highlighted that phasic voltage variation of QRS complexes in V4-6 observed in left-sided pneumothorax are due to the heart’s periodic movement within the thorax caused by respiration [1].

## Case presentation

A 26-year-old young, slim male patient of medium build (height 176 cm, weight 72 kilograms) presented with sudden-onset central chest pain for 3 hours, worse on lying down, leaning forward, deep inspiration and expiration. He did not have any features suggestive of Marfan syndrome such as long arms and legs (arachnodactyly), high arched palate, crown to pubic symphysis to height ratio and hypertelorism. He had a mild dry cough and denied fever, trauma, travel history, and deep-sea diving. He denied any shortness of breath and had worsening chest pain on breathing and sitting up. He did not have any nausea or vomiting. He was a lifelong non-smoker and social drinker. He was not on any regular medications and did not have any past medical history of note. On arrival, his initial vitals were blood pressure 114/68, heart rate 68 bpm, temp 37.6C, respiratory rate (RR) 24 and SpO_2_ 97%. Physical examination was unremarkable apart from reduced air entry on the left side and normal heart sounds with no audible pericardial rub. Laboratory tests were all within normal limits; both troponin-T and D-dimer were negative (Table 1).

**Table 1:** Laboratory test results ALT, alanine transaminase.

Test	Value	Reference range
Sodium	140 mmol/L	(133-146)
Potassium	3.9 mmol/L	(3.5-5.3)
Urea	4.5 mmol/L	(2.5-7.8)
Creatinine	94 umol/L	(59-135)
Troponin T	11 ng/L	(<14)
ALT	13 U/L	(<50)
Total bilirubin	13 umol/L	(0-21)
Alkaline phosphatase	113 U/L	(30-130)
C-reactive protein	<1 mg/L	(0-5)
Albumin	46 g/L	(35-50)
Neutrophil count	5.99 × 10^9^/L	(1.7-7.5)
Platelet count	197 × 10^9^/L	(150-400)
White cell count	8.9 × 10^9^/L	(4.0-11.0)
Haemoglobin	146 g/L	(130-180)
D-dimer	146 ng/mL	(<243)

**Figure 1:**
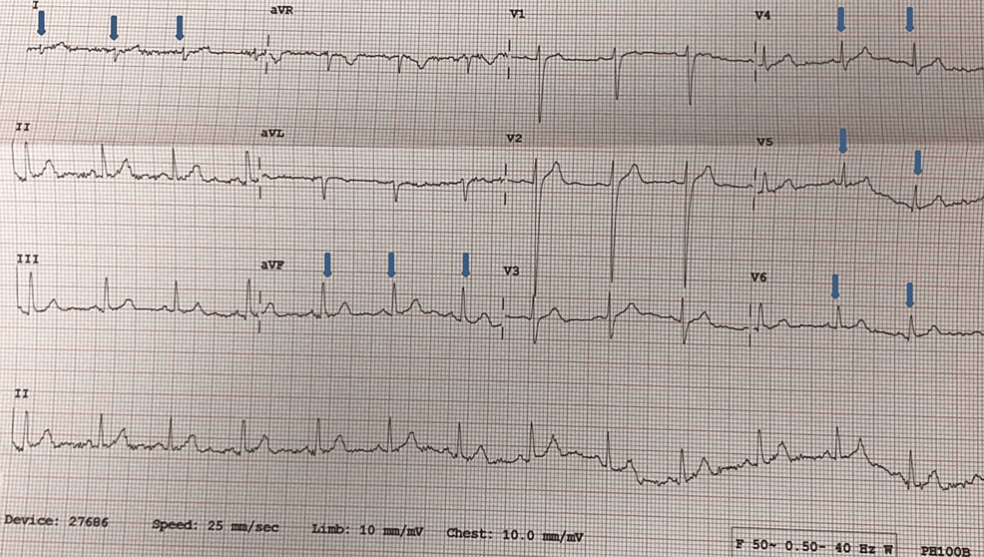
Electrocardiogram showing right axis deviation (negative QRS in lead 1 and positive QRS in lead AVF) and small-amplitude QRS complexes and diminished-amplitude R waves in precordial leads V4-V6 as shown by arrows.

**Figure 2:**
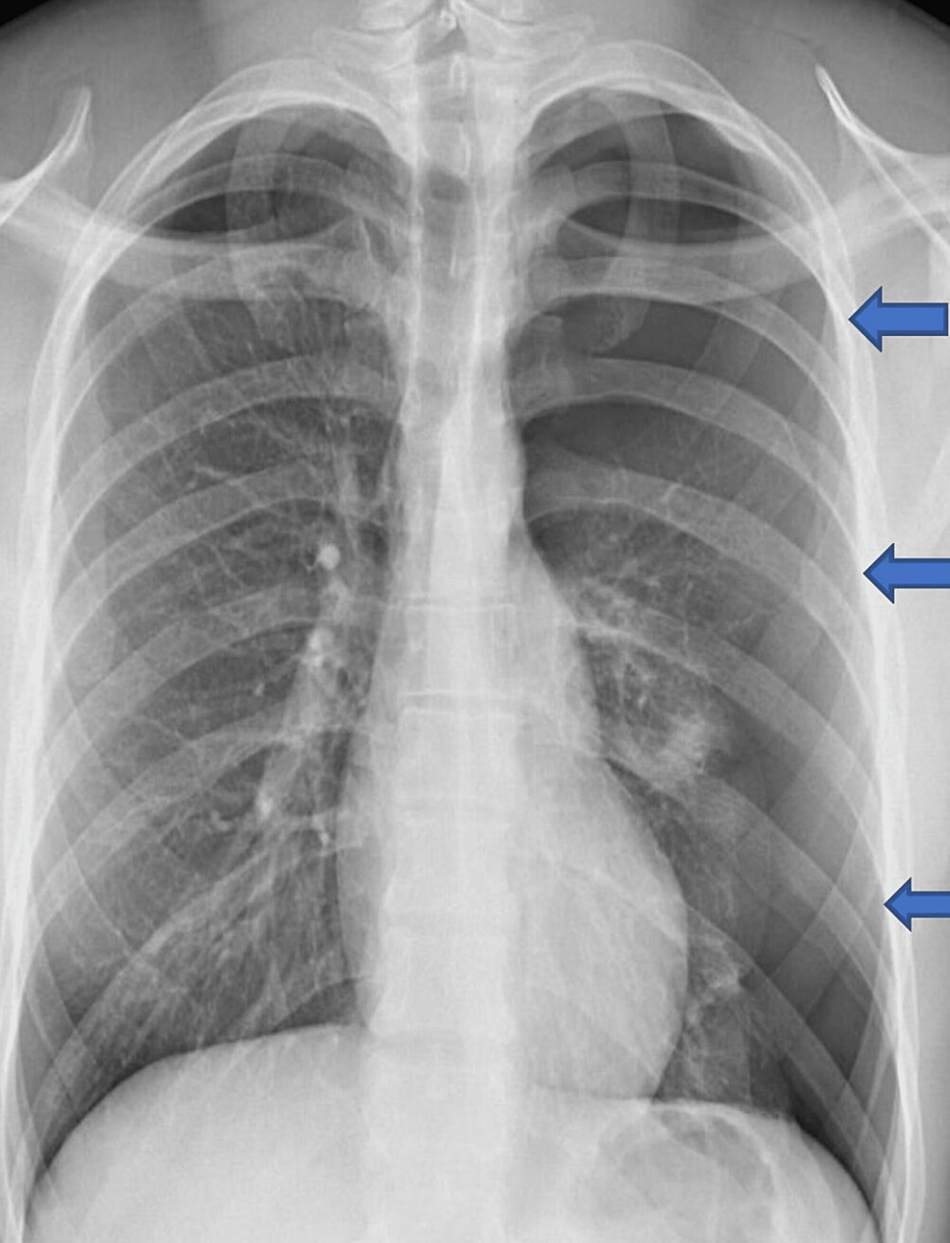
Chest radiography showing left-sided pneumothorax as shown by the arrows.

**Figure 3:**
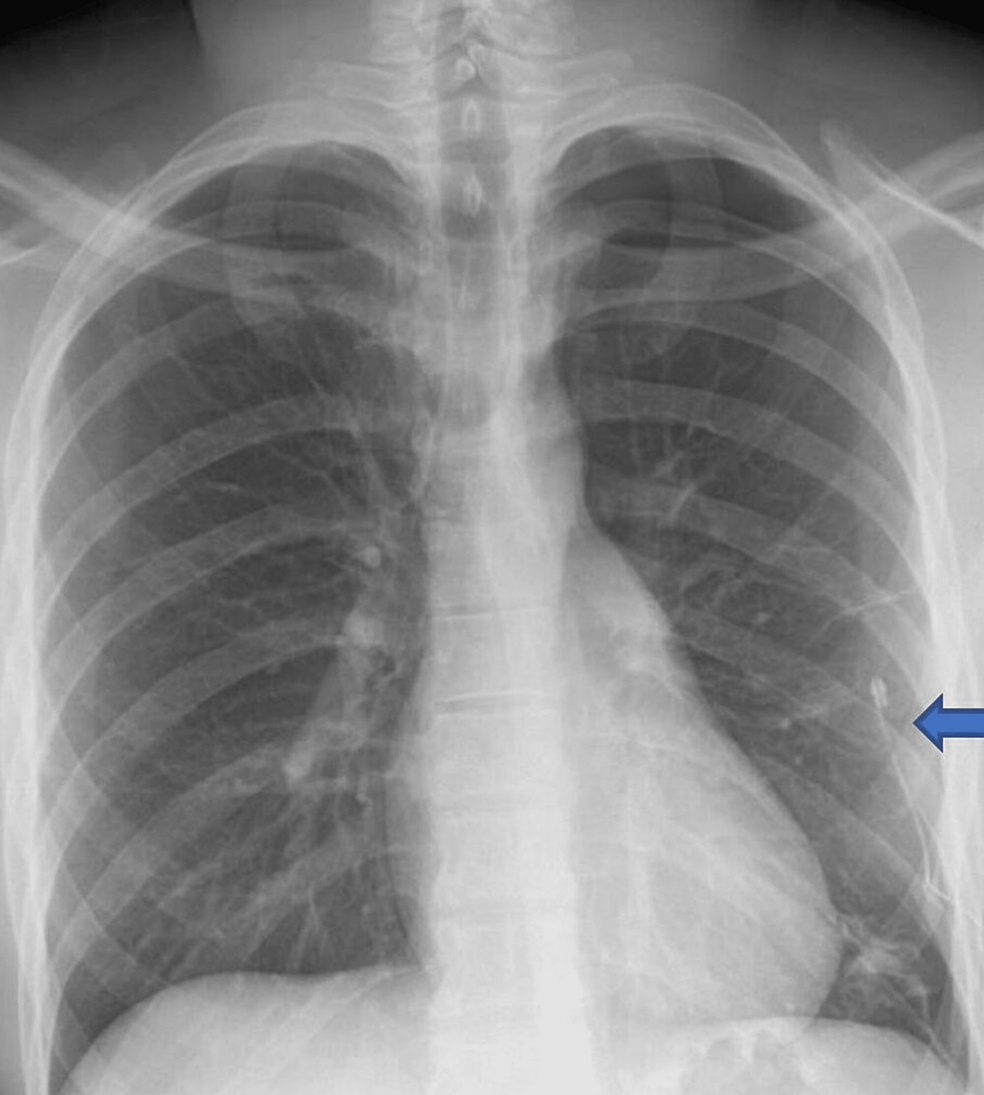
Chest radiography post drain insertion showing complete resolution of the left-sided pneumothorax and chest drain tip shown by the arrow.

ECG showed small QRS complexes and diminished R wave amplitude in lateral leads 1, AVL, V4-V6 and RAD, which is confirmed by downgoing QRS in lead 1 and upgoing QRS complexes in lead AVF (Figure 1). The ECG changes are commonly observed in left-sided pneumothorax and completely resolve with the resolution of pneumothorax. In addition to the above, T waves inversion is another common finding observed in patients with left-sided pneumothorax along with RAD, diminished amplitude R waves and small-amplitude QRS complexes which is believed to be due to insulated air trapped between the pleural cavity and the chest wall. Chest radiography showed significant left-sided pneumothorax and the patient had an emergency ultrasound-guided chest drain inserted resulting in a reduction in the size of the pneumothorax (Figures 2, 3).

The patient was admitted to the respiratory ward and the pneumothorax showed complete resolution within 48 hours. Repeat ECG showed normal sinus rhythm, resolution of RAD, normal-amplitude R waves and normal-sized QRS complexes in the precordial leads. The patient did not show any haemodynamic compromise during admission and was discharged home with outpatient respiratory follow-up after four days of hospital stay. This case is important as the ECG changes observed in our case have previously been reported which include small QRS amplitudes in precordial leads and RAD and sometimes these findings can mimic pericardial effusion. Therefore, these findings can be particularly important for emergency physicians to be aware of in young patients presenting with chest pain without any shortness of breath.

## Discussion

Pneumothorax is defined as abnormal air trapped within the pleural space between the lungs and the chest wall and the patients may present with chest pain, shortness of breath, tachycardia, tachypnoea and chest tightness. PSP occurs mainly in adolescents and can mimic a cardiac event [2]. PSP occurs in approximately seven per 100,000 men and one per 100,000 women per year. There are several documented electrocardiographic findings in patients with spontaneous pneumothorax which include axis deviation, bundle branch block, T wave inversion and small-amplitude QRS complexes [2,3]. The electrocardiographic features in left-sided pneumothorax have been more clearly described compared to right-sided pneumothorax in the literature [2-5]. RAD in ECGs can also be found in patients with pulmonary embolism and exacerbation of lung disease [4]. Few case reports have reported ECG features of RAD and inverted p waves in patients with spontaneous pneumothorax which resolved completely with the resolution of the pneumothorax [3,6-8].

A retrospective study of 57 patients with PSP underwent cardiac evaluation and the study highlighted key ECG features in these patients [2]. Forty-nine patients were males, and the median age was 16 years. Of these, 56 patients had a unilateral pneumothorax and one patient had a bilateral pneumothorax. The main presenting complaint was dyspnoea and chest pain in 67% and 33% of patients, respectively. Small, medium and large unilateral pneumothorax was observed in 9, 30 and 17 patients, respectively, whereas one patient had bilateral pneumothorax as mentioned before. ECGs were abnormal in 12 (21%) patients and the common ECG findings were ST elevation in five patients, inverted T wave in two patients, incomplete right bundle branch block in two patients, poor R wave progression, left axis deviation and low QRS voltage in one patient each and troponin was normal in all these patients. ECG changes among the paediatric patients with PSP were found in 21% of patients and the common ECG findings included ST-segment elevation, inverted or flattened T wave, incomplete right bundle branch block, poor R wave progression, left axis deviation and low QRS voltage.

Several other studies have reported ECG findings in patients with left-sided PSP [5,9,10]. Waltson et al. observed four ECG changes in patients with left-sided pneumothorax including rightward QRS deviation, precordial T wave inversion, decreased QRS amplitude and diminution of precordial R voltage. They postulated that the ECG changes such as the rightward shift of the QRS axis may be due to the rotation of the heart around its longitudinal axis and increase in pulmonary vascular resistance leading to right ventricular dilatation [9]. Similarly, the diminution of the precordial R voltage may be due to reduced conductance of the electrical impulse by retrosternal free air and posterior displacement of the heart. Krenke et al. reviewed the ECGs of 40 patients with PSP and noticed T wave inversion in only one patient; however, none of these patients had ST-segment elevation [4]. Klin et al. reported ST segment elevation in five patients and four out of these five patients had right-sided pneumothorax [2]. Very few case reports of ST-segment elevation associated with pneumothorax have been published and these are mainly seen in older patients with a history of coronary artery disease (CAD). However, Shiyovich et al. published a case report of a young patient presenting with ST-segment elevation with left-sided primary pneumothorax and few authors have contributed these findings to transient coronary ischaemia to be secondary to hypoxia and hypotension caused by mediastinal displacement [10]. Our patient presented with large left-sided PSP and had small-amplitude QRS complexes and RAD.

It is worth mentioning here that the ECG changes seen in pneumothorax are slightly different than those seen in MI, pericardial effusion and pulmonary embolism. For example, ECG in left-sided pneumothorax can present with RAD and small QRS amplitudes in precordial leads secondary to mechanisms discussed above. Kozelj et al. (1997) described a case of a 43-year-old man with a left-sided pneumothorax with a frontal plane axis of 74 degrees and phasic voltage alternation [11]. Soltani et al. (2009) described another case of a 46-year-old man with ECG features of RAD and phasic voltage alternation secondary to a left-sided pneumothorax [12]. Walston et al. described that left-sided pneumothorax can show features of RAD, diminution of precordial R voltage and a decrease in QRS amplitude and similar features were seen in our patient confirming the fact that patients with left-sided pneumothorax can present with certain ECG features. Feldman and January (1984) described the small QRS amplitudes observed in left-sided pneumothorax to be secondary to air insulating the chest wall, rather than cardiac rotation, dilatation or displacement as a mechanism of the ECG change [13]. 

## Conclusions

In conclusion, ECG changes are not uncommon in patients presenting with PSP, and patients presenting with chest pain and dyspnoea should have ECG recorded as part of the initial work-up. Physicians should pay attention to ECG changes in patients and this can be helpful to guide us towards non-cardiac causes such as pulmonary embolism, pneumothorax and chronic lung conditions. ECG changes are more common in left-sided pneumothorax compared to the right and the size of the pneumothorax does not have any influence on the ECG changes. The most common ECG findings in patients with primary spontaneous left-sided pneumothorax include right axis deviation, low-amplitude QRS complexes and diminished-amplitude R waves in precordial leads.
